# Prevalence and Influencing Factors of Thyroid Dysfunction in HIV-Infected Patients

**DOI:** 10.1155/2016/3874257

**Published:** 2016-04-20

**Authors:** Shujing Ji, Changzhong Jin, Stefan Höxtermann, Wolfgang Fuchs, Tiansheng Xie, Xiangyun Lu, Haibo Wu, Linfang Cheng, Adriane Skaletz-Rorowski, Norbert H. Brockmeyer, Nanping Wu

**Affiliations:** ^1^State Key Laboratory for Diagnosis and Treatment of Infectious Diseases, The First Affiliated Hospital, School of Medicine, Zhejiang University, Hangzhou 310003, China; ^2^Collaborative Innovation Center for Diagnosis and Treatment of Infectious Diseases, Hangzhou 310003, China; ^3^Department of Dermatology and Allergology, St. Josef-Hospital, Ruhr-University Bochum, 44791 Bochum, Germany

## Abstract

Thyroid dysfunction is more common in human immunodeficiency virus (HIV) patients. But the effects of highly active antiretroviral therapy (HAART) and hepatitis B/C virus (HBV/HCV) coinfection on thyroid function is unclear. We retrospectively reviewed the data of 178 HIV patients and determined the prevalence of thyroid dysfunction and the relationship between thyroid hormone levels, CD4 cell count, HIV-1 duration, HAART duration/regimens, and HBV/HCV coinfection. Of the 178 patients, 59 (33.1%) had thyroid dysfunction, mostly hypothyroidism. Thyroid dysfunction was significantly more frequent in the HAART group (41/104, 39.4%) than in the HAART-naïve group (18/74, 24.3%; *P* < 0.05). The mean CD4 cell count was significantly lower in patients with hypothyroidism (372 ± 331/*μ*L) than in the other patients (*P* < 0.05). The FT4 level was significantly lower in the HAART group than in the HAART-naïve group (1.09 ± 0.23 versus 1.20 ± 0.29 pg/mL, *P* < 0.05). FT3/FT4 levels were negatively related to HIV duration and FT3 levels were positively related to CD4 cell (*P* < 0.05). HBV patients had lower FT3 levels, while HCV patients had higher FT3 and FT4 levels (*P* < 0.05). Thyroid dysfunction is more common in HIV patients on HAART, mainly manifested as hypothyroidism. FT3/FT4 levels are correlated with HIV progression. HBV/HCV coinfection increases the probability of thyroid dysfunction.

## 1. Introduction

Human immunodeficiency virus (HIV) infection is characterized by decreased CD4 cell count and immunodeficiency, leading to opportunistic infections (OIs) and tumors [1]. In recent years, increasing number of patients with HIV infection are able to survive for long periods because of the extensive application of highly active antiretroviral therapy (HAART) for the repression of viral replication as well as because of the emergence of new medicines and therapeutic regimens. Many nonacquired immune deficiency syndrome- (AIDS-) related diseases now primarily account for the disease burden in patients with HIV infection. Abnormalities of the endocrine function of the pituitary, thyroid, adrenals, gonads, and pancreas and in metabolism are common in patients infected with HIV and are becoming the main conditions influencing the long-term quality of life in HIV-infected patients [2–8]. Some studies have reported complications such as hypertriglyceridemia and hypercholesterolemia, lipodystrophy and lipoatrophy, glucose intolerance and type 2 diabetes mellitus, gonadal dysfunction, and osteopenia and osteoporosis during HAART [2, 9–13]. Thyroid hormone, an important hormone regulating metabolism, can also be affected by HIV infection. Numerous studies have reported that the incidence of thyroid dysfunction is much higher (about 36%-37%) in patients infected with HIV than in the general population [14, 15]. However, other researchers have suggested that the morbidity of overt thyroid dysfunction in patients infected with HIV is similar to that in the general population [2–8, 15–21]. Therefore, further research into the prevalence of thyroid dysfunction in patients infected with HIV is required.

Thyroid dysfunction reduces the quality of life of patients infected with HIV. Overt hypothyroidism leads to the insidious onset of fatigue, weakness, dry skin, cold intolerance, slowed mentation, constipation, hoarse voice, paresthesia, bradycardia, and delayed relaxation of tendon reflexes. Overt hyperthyroidism is characterized by irritability, heat intolerance, sweating, warm moist skin, palpitations, tachycardia, fatigue, weight loss with increased appetite, diarrhea, tremor, muscle weakness, hyperreflexia, and lid retraction. The consequences of subclinical hyperthyroidism include reduced bone mineral density and an increased risk of atrial fibrillation, the risk of which is proportional to the degree of thyroid hyperfunction [15]. Furthermore, subclinical hyperthyroidism may precede overt hyperthyroidism [20, 22]. It is unclear why HIV-infected patients are susceptible to thyroid dysfunction, but HIV infection is regarded as a crucial factor. Furthermore, the influence of HIV infection on thyroid function changes with the course of the disease. Asymptomatic, subtle abnormalities of thyroid function tests have been described in a small minority of patients with stable HIV infection [2, 16, 17]. With the progression of the disease, a pattern of sick euthyroid syndrome may develop. The most frequent abnormalities in thyroid function tests are those associated with subclinical hypothyroidism [2, 15, 22–24]. The medications used to treat HIV infection are also a vital factor leading to abnormalities in thyroid function. Some reports have indicated that HAART increases the probability of thyroid dysfunction. Stavudine has been suggested to directly affect the production and/or metabolism of thyroid hormones [2, 14, 22, 24]. Prolonged treatment with stavudine contributes to a decrease in free thyroxine (FT4) level [2]. Bongiovanni et al. showed that the accumulation of the daily intake of stavudine and lamivudine is responsible for the occurrence of hypothyroidism [1]. Beltran et al. found that stavudine and low CD4 cell counts were associated with hypothyroidism [22]. Parsa and Bhangoo determined that stavudine was most often associated with subclinical hypothyroidism [20, 25–27]. Madeddu et al. reported that thyroid-stimulating hormone (TSH) was negatively correlated with CD4 cell count nadir and positively correlated with HAART duration [2]. They also reported that there was no significant correlation between free tri-iodothyronine (FT3), FT4, age, duration of HIV infection, duration of HAART, CD4 cell count, and CD4 cell count nadir [2]. Besides HAART, treatment regimens including rifampicin or immunomodulatory agents have often been reported to alter thyroid hormone levels with clinical effects in patients with HIV-related diseases [2, 28, 29]. Furthermore, some researchers have suggested that hepatitis B virus (HBV) and/or hepatitis C virus (HCV) coinfection increases the risk of thyroid dysfunction in HIV-infected patients, and according to Beltran et al., HCV coinfection is a risk factor for hypothyroidism [22]. Thus far, the relationship between thyroid dysfunction and HIV infection is obscure, especially, with respect to the state of HIV and HAART. In addition, the influence of drugs other than stavudine on thyroid function, such as protease inhibitors (PIs), integrase inhibitors (IIs), and CCR5 antagonists, has not yet been reported. In this study, we analyzed the thyroid function of 178 patients infected with HIV, of which 104 were on HAART. We found that thyroid dysfunction was common in patients infected with HIV, especially, in patients on HAART, and hypothyroidism accounted for the majority of cases of thyroid dysfunction. Moreover, the levels of FT3 and FT4 were related to disease progression. PIs and IIs had no significant influence on thyroid function, and hyperthyroidism frequently occurred in patients with HCV coinfection.

## 2. Materials and Methods

### 2.1. Subjects

We analyzed the clinical information of 178 HIV patients who were treated at St. Josef-Hospital, Ruhr-University Bochum, between June and September 2014. In all patients, HIV infection was diagnosed by the detection of HIV antibodies in blood using enzyme-linked immunosorbent assay (ELISA) and western immunoblot analysis. Disease stages of HIV-1 patients were diagnosed based on CDC revised classification system for HIV infection and expanded surveillance case definition for AIDS among adolescents and adults (1993) [30]. Of the 178 patients, 104 were treated with HAART, while 74 were HAART-naïve. We recorded the age, sex, transmission route and duration of HIV infection, and the duration and type of therapeutic regimens used. The course of the disease was defined as the duration of HIV infection calculated from the first HIV-positive test result [22], and the course of HAART was calculated from the day on which HAART was started. Patients were systematically screened for coinfection with HCV or HBV.

### 2.2. Laboratory Tests

CD4 cell count was measured by flow cytometry (FACSCantoII, Becton Dickinson, Franklin Lakes, NJ, USA), and plasma HIV load (HIV RNA) was determined using a standardized reverse transcriptase-polymerase chain reaction assay (Cobas Amplicor HIV-1 Monitor Test, version 1.5, Ultrasensitive specimen preparation, Roche Diagnostic Systems Inc., Branchburg, NJ, USA). The lower limit of detection in plasma was 50 HIV-1 RNA copies/mL. HCV infection was detected by measuring antibodies to HCV in serum samples by using ELISA (Ortho HCV 3.0 ELISA test, Ortho Clinical Diagnostic, Amersham, Buckinghamshire, UK) and an immunoblot assay (SIA, Chiron RIBA, Chiron Corporation, Emeryville, CA, USA). HBV infection was detected by measuring the hepatitis B surface antigen level using an enzyme-linked fluorescent assay (Biomerieux, Lyon, France). Serum TSH level was measured by a one-step radioimmunometric assay (IRMA-mat, Byk-Sangtec Diagnostica, Dietzenbach, Germany), and serum FT3 and FT4 levels were measured using radioimmunological assays (Amerlex MAB, Ortho Clinical Diagnostics, Milan, Italy).

### 2.3. Definition of Thyroid Function Abnormalities

The normal ranges of TSH, FT3, and FT4 were 0.27–4.20 *μ*IU/mL, 2.00–4.40 pg/mL, and 0.93–1.70 pg/mL, respectively. Overt hypothyroidism was defined as a low FT3 and/or FT4 level, with/without a high TSH level. Overt hyperthyroidism was defined as a high FT3 and/or FT4 level, with/without a low TSH level. Subclinical hypothyroidism was defined as a high TSH level with normal FT3 and FT4 levels, while subclinical hyperthyroidism was defined as a low TSH level with normal FT3 and FT4 levels. All the clinical and laboratory data was analyzed for patients with thyroid function abnormalities when they were first diagnosed with thyroid dysfunction in this cross-sectional study.

### 2.4. Statistical Analysis

Statistical analysis was performed using the SPSS (version 20; IBM Corp., Armonk, NY, USA). Numerical/continuous variables were reported as mean ± standard deviation, and qualitative/categorical variables were expressed as the number of cases and percentages. Group means were compared using the independent-samples *t*-test, *F*-test, and *χ* ^2^ test. The Pearson correlation coefficient was used to assess the association between numerical/continuous variables and Spearman correlation coefficient was used to assess the association between discrete variables. All comparisons were two-sided, and *P* < 0.05 was the cutoff value for statistical significance. Univariate and multivariate logistic regression analyses were done, and probability for stepwise was 0.1 to entry and 0.15 to removal.

## 3. Results

### 3.1. Clinical Information of HIV-Infected Patients

The mean age of the 104 HIV-infected patients treated with HAART was 50.18 ± 10.19 years, and the mean duration of HAART was 7.42 ± 5.2 years. The mean age of the 74 HAART-naïve patients was 46.68 ± 11.64 years. There were multiple routes of transmission, and 69 (66.3%) patients on HAART and 47 (63.5%) HAART-naïve patients were infected via homosexual acts. The duration of HIV infection was significantly longer in the HAART patients (14.81 ± 7.21 years) than in the HAART-naïve patients (7.81 ± 5.26 years; *P* < 0.05). The viral load was significantly lower in the HAART group [10E (1.06 ± 1.64)] than in the HAART-naïve group [10E (4.12 ± 1.82)] (*P* < 0.05). CD4 cell count was higher in the HAART group (547 ± 305/*μ*L) than in the HAART-naïve group (392 ± 319/*μ*L; *P* < 0.05; Table 1). CD4 cell count nadirs were not significantly different between HAART and HAART-naïve groups (370.96 ± 225.25 versus 293.26 ± 220.45/*μ*L, *P* = 0.499, Table 1). There were many more patients diagnosed with CDC class 1 in the HAART group than in HAART-naïve group (57 (54.8%) versus 19 (26.0%)) (*P* < 0.05; Table 1). There were totally 34 (19.1%) patients diagnosed with OIs, of whom 22 were diagnosed with herpes infection and 7 were diagnosed with thrush.

### 3.2. Prevalence of Thyroid Dysfunction in HIV-1-Infected Patients

**Figure 1:**
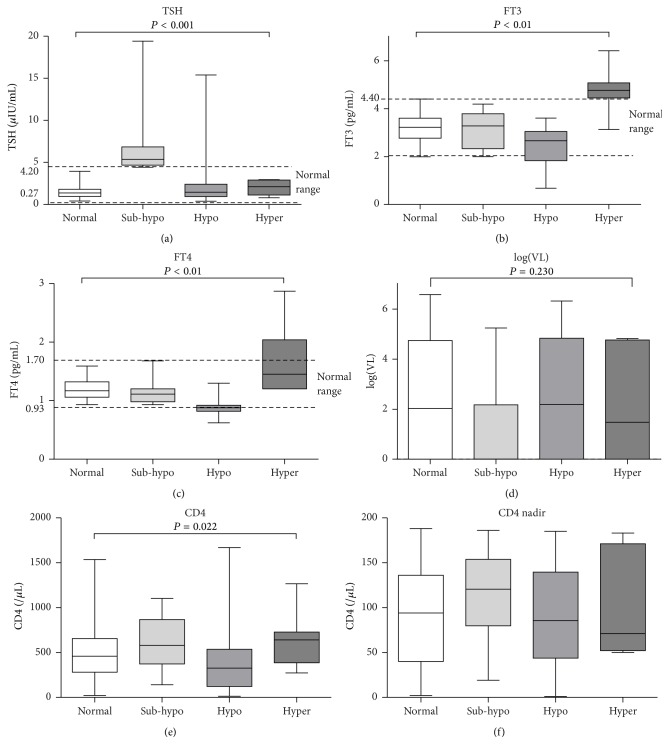
Thyroid hormone levels, HIV loads, and CD4 cell counts in patients with different thyroid function. FT3, free tri-iodothyronine; FT4, free thyroxine; TSH, thyrotropin; VL: viral load. Normal: normal thyroid function; sub-hypo: subclinical hypothyroidism; hypo: overt hypothyroidism; hyper: overt hyperthyroidism. The horizontal lines represent mean values, the boxes represent standard deviations, and the whiskers represent ranges. The dashed lines represent the normal ranges of thyroid hormones. The *P* values in the figure were acquired using *χ* ^2^ tests of the whole sample and not from comparisons between any two means.

Of the 178 study patients, 59 (33.1%) were diagnosed with thyroid dysfunction, including 12 (6.6%) patients with subclinical hypothyroidism, 40 (22.1%) with overt hypothyroidism, and 7 (3.9%) patients with overt hyperthyroidism. There were not any patients with subclinical hyperthyroidism. 41 of the patients with overt thyroid dysfunction had normal TSH levels, including 34 with overt hypothyroidism and 7 with overt hyperthyroidism. Thyroid dysfunction was significantly more frequent in patients on HAART than in HAART-naïve patients (*P* < 0.05). Of the 104 patients on HAART, 41 (39.4%) had thyroid dysfunction, including 9 (8.7%) with subclinical hypothyroidism, 28 (26.9%) with overt hypothyroidism, and 4 (3.8%) with overt hyperthyroidism. Of the 74 HAART-naïve patients, 18 (24.3%) patients had thyroid dysfunction, including 3 (4.1%) with subclinical hypothyroidism, 12 (16.2%) with overt hypothyroidism, and 3 (4.1%) with overt hyperthyroidism. Hypothyroidism was the most frequent dysfunction. Thyroid dysfunction was significantly more frequent in the HAART group than in the HAART-naïve group (*P* = 0.006, Table 1). The mean thyroid hormone (TSH, FT3, and FT4) levels in patients with thyroid dysfunction and those with normal thyroid function are shown in Figure 1. The mean CD4 cell count was significantly lower in patients with overt hypothyroidism (372 ± 331/*μ*L) than in patients in the other groups shown in Figure 1 (*P* < 0.05). But the counts of CD4 cell nadir were similar among the four groups of patients.

We found no different incidence of thyroid dysfunction in patients of different CDC classes. FT3 level was higher in patients of CDC class 1 (3.32 ± 0.67 pg/mL) than in patients of CDC class 3 (2.55 ± 0.86 pg/mL, *P* < 0.05). Neither TSH nor FT4 levels were different among patients of the three CDC classes. We also compared the incidence of thyroid dysfunction between patients with and without OIs. No different incidence of thyroid dysfunction and different levels of thyroid hormones were found in patients with OIs compared with those without OIs.

### 3.3. Effect of HAART on Thyroid Hormone Levels

**Figure 2:**
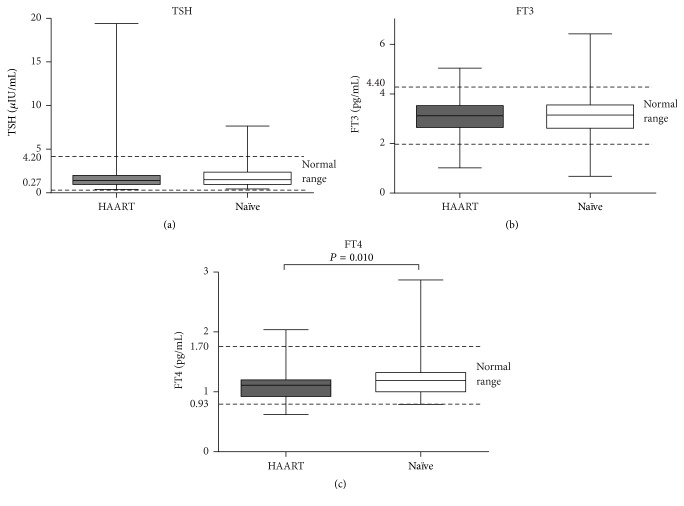
Thyroid hormone levels in patients on HAART and in HAART-naïve patients. FT3, free tri-iodothyronine; FT4, free thyroxine; TSH, thyrotropin; HAART, HIV patients receiving highly active antiretroviral therapy (HAART); Naïve: HIV patients who were HAART-naïve. The horizontal lines represent mean values, the boxes represent standard deviations, and the whiskers represent ranges. The dashed lines represent the normal ranges of thyroid hormones. *P* values > 0.05 in pairwise comparisons are not shown in the figure.

The FT3 and FT4 levels in the HAART group were 3.09 ± 0.73 pg/mL and 1.09 ± 0.23 pg/mL, respectively. Both these values were lower than the levels in the HAART-naïve patients (3.12 ± 0.86 pg/mL and 1.20 ± 0.29 pg/mL, resp.); however, only the difference between the FT4 levels was significant (*P* < 0.05). The TSH level was higher in the HAART group (2.09 ± 2.53 *μ*IU/mL) than in the HAART-naïve group (1.87 ± 1.37 *μ*IU/mL, *P* = 0.446; Figure 2).

We also found that the levels of FT3 and FT4 were negatively correlated with the duration of HIV infection and positively correlated with CD4 cell count in the HAART and HAART-naïve groups (*P* < 0.05), although the strength of the correlation was weak (Table 2). The FT3 and FT4 levels were not significantly related to HAART duration, viral load, CD4 cell count nadir, or any other parameters. No relevance was found between TSH levels or any of the above disease parameters.

**Figure 3:**
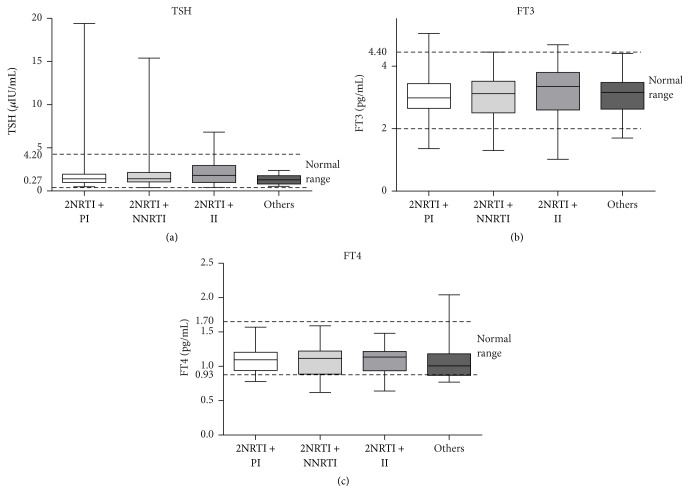
Thyroid hormone levels in HIV-infected patients on different therapies. FT3, free tri-iodothyronine; FT4, free thyroxine; TSH, thyrotropin; NRTI, nucleoside reverse transcriptase inhibitor; PI, protease inhibitor; NNRTI, nonnucleoside reverse transcriptase inhibitor; II: integrase inhibitor; others: other therapies (PI + II, 3 PIs, etc.). The horizontal lines represent mean values, the boxes represent standard deviations, and the whiskers represent ranges. The dashed lines represent normal ranges of thyroid hormones. *P* values > 0.05 in pairwise comparisons are not shown in the figure.

We further studied the effect of different HAART regimens on thyroid function. As none of patients were taking stavudine, we divided the 104 patients with HAART into four subgroups: 28 (26.9%) patients taking two nucleoside reverse transcriptase inhibitors (NRTIs) + PI; 40 (38.5%) patients taking two NRTIs + nonnucleoside reverse transcriptase inhibitors (NNRTIs); 21 (20.2%) patients taking two NRTIs + II; and 15 (14.4%) patients following other therapeutic regimens. Thyroid hormone levels did not differ among these four subgroups, indicating that there were no significant differences in the effects of PIs, IIs, and NNRTIs on thyroid function (Figure 3).

### 3.4. Effect of HBV/HCV Coinfection on Thyroid Function

**Figure 4:**
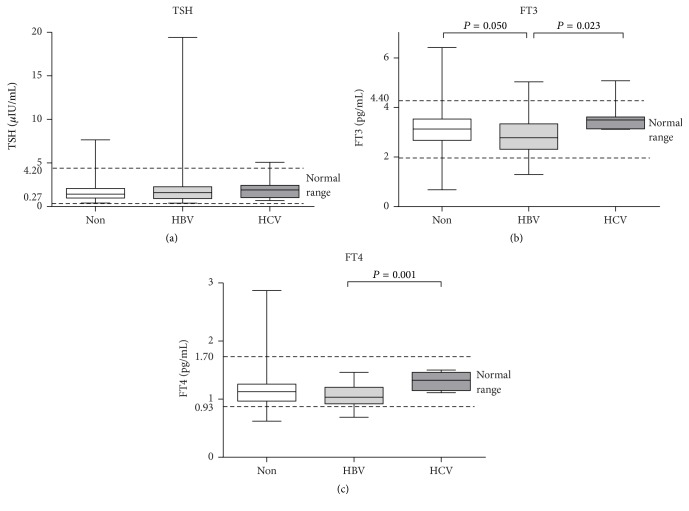
Thyroid hormone levels in HIV-infected patients with or without HBV/HCV coinfection. FT3, free tri-iodothyronine; FT4, free thyroxine; TSH, thyrotropin; HBV, hepatitis B virus (HBV) coinfection; HCV, hepatitis C virus (HCV) coinfection; Non: no HBV/HCV coinfection. Thyroid hormone of the first line of the picture belongs to all of the patients; thyroid hormone of the second line belongs to patients with normal thyroid function; thyroid hormone of the third line belongs to patients with thyroid dysfunction. The horizontal lines represent mean values, the boxes represent standard deviations, and the whiskers represent ranges. The dashed lines represent the normal ranges of thyroid hormones. *P* values > 0.05 in pairwise comparisons are not shown in the figure.

HBV/HCV coinfection has been reported to lead to thyroid dysfunction, especially hypothyroidism [22]. We therefore divided the patients into three groups depending on the infectious agent: no coinfection (139 patients), HBV coinfection (31 patients), and HCV coinfection (8 patients). There were 43 (30.9%) patients diagnosed with thyroid dysfunction in patients without coinfection, 14 (45.2%) patients diagnosed with thyroid dysfunction in patients coinfected with HBV, and 2 (25%) patients diagnosed with thyroid dysfunction in patients coinfected with HCV. The FT3 and FT4 levels were significantly higher in the HCV patients (3.60 ± 0.64 pg/mL, 1.31 ± 0.15 pg/mL, resp.) than the HBV patients (2.82 ± 0.85 pg/mL, 1.06 ± 0.18 pg/mL, resp., *P* < 0.05). The FT3 level was higher in patients without coinfection (3.13 ± 0.76 pg/mL) than in those with HBV coinfection (2.82 ± 0.85 pg/mL, *P* < 0.05). No differences in TSH levels were found among these three groups of patients (Figure 4).

### 3.5. Univariate and Multivariate Logistic Regression Fitted for Thyroid Dysfunction

To control for possible confounding variables, we conducted univariate and multivariate logistic regression analysis. Univariate logistic regression revealed that sex was the only risk factor for thyroid dysfunction and the odds ratio (OR) and 95% confidence interval (CI) were 2.082 (0.884, 4.903) (*P* = 0.093) (Table 3). Females were at higher risk to suffer from thyroid dysfunction. Although duration of HIV infection and CD4 cell count nadir were related to thyroid dysfunction and their ORs and 95% CIs were 1.047 (1.003, 1.093) (*P* = 0.037) and 0.999 (0.997, 1.000) (*P* = 0.061) (Table 3), they were not risk factors for thyroid dysfunction. Multivariate logistic regression indicated that ORs and 95% CIs of sex, duration of HIV infection, and CD4 nadir were 2.231 (0.919, 5.414) (*P* = 0.076), 1.057 (1.011, 1.105) (*P* = 0.014), and 0.998 (0.997, 1.000) (*P* = 0.041), which were consistent with univariate logistic regression analysis. Transmission routes, HBV or HCV coinfection, viral load, CD4 cell count, and CDC classification had no significant relation to thyroid dysfunction.

## 4. Discussion

In our study, 33.1% of HIV-infected patients were diagnosed with thyroid dysfunction, which is significantly higher than the rate reported by Beltran et al. (16%) [22]. This difference may be attributable to the longer duration of HIV infection in our study, considering that FT3 and FT4 levels were negatively correlated with duration of HIV infection. The mean duration of HIV infection in the HAART and HAART-naïve groups was 14.81 ± 7.21 years and 7.81 ± 5.26 years, respectively. Patients with longer HIV duration had lower FT3 and FT4 levels, indicating a higher possibility of hypothyroidism. Thyroid dysfunction was more common in the HAART group than in the HAART-naïve group, and clinical hypothyroidism was the most common thyroid dysfunction in HIV-infected patients, which is consistent with the findings of Beltran et al. [22]. However, Madeddu et al. reported that subclinical hypothyroidism was the most frequent thyroid dysfunction in HIV patients treated with HAART [2, 22–24, 31]. While Hoffmann et al. concluded that the probability of clinical hypothyroidism in HIV-infected patients was similar to that in the general population, the probability of abnormal thyroid hormone test results was higher in the former group [1–3, 15–21]. Many patients are diagnosed with subclinical hypothyroidism during antiretroviral therapy [2, 15, 22–24, 31]. Graves' disease has often been reported during immune reconstitution [15]. The above differences in the incidence of thyroid dysfunction in HIV-infected patients may be related to differences in disease course, criteria of hypothyroidism, subclinical hypothyroidism, and so forth.

As far as we know, many cases of thyroid dysfunction in HIV-infected patients are attributable to HAART. Many longitudinal studies have demonstrated that HAART plays an important role in thyroid function abnormality appearance. A cohort study by Nelson et al. demonstrated that hypothyroidism was most commonly associated with PI and hyperthyroidism with NNRTI, especially efavirenz [32]. HAART could dramatically affect plasma HIV RNA and increased memory and naïve CD4 cell; in the meantime, infectious or self-antigens might be produced and immune reconstitution inflammatory syndrome (IRIS) occurred to act against the antigens [33]. IRIS might lead to autoimmunity in HIV-1-positive patients [34] and the majority of the hypothyroidism was regarded as a result of autoimmune etiology [35]. We therefore analyzed the effects of HAART on thyroid function and found that the FT4 level was significantly lower in the HAART group than in the HAART-naïve group. However, no significant differences in thyroid hormone levels were found among patients on different HAART regimens, indicating that there were no significant differences in the effects of PIs, IIs, and NNRTIs on TSH, FT3, and FT4 levels. A predominant and definite action by a single pharmaceutical product included in the HAART regimen that could affect thyroid function has not been consistently ascertained. Stavudine has been suggested to directly affect the production and/or metabolism of thyroid hormones [2, 14, 22, 24, 27]. A follow-up experiment by Madeddu et al. illustrated that prolonged stavudine treatment significantly decreased FT4 levels [2]. While Beltran et al. found on multivariate analysis that stavudine treatment and low CD4 cell counts were associated with hypothyroidism [22]. In addition, Madeddu et al. [2] found no significant difference in the levels of FT3, FT4, and TSH between patients treated with PIs and those treated with non-PI drugs, but they observed that FT4 levels were lower in both these groups than in HAART-naïve patients, which is consistent with our findings. The role of HAART in thyroid function abnormalities development should be better clarified in a long follow-up prospective study.

FT3 and FT4 levels have been reported to be related to the state of HIV infection and are potential biomarkers of HIV progression. Beltran et al. reported that compared with HIV-infected patients with normal thyroid function, patients with hypothyroidism were older, had HIV infection for a longer duration, had lower CD4 cell counts, and were more likely to have HCV coinfection and have been treated with HAART [22]. On multivariate analysis, however, the same authors found that only stavudine treatment and low CD4 cell count were statistically associated with hypothyroidism [22]. Madeddu et al. found that TSH levels were negatively correlated with CD4 cell count nadir and positively correlated with HAART duration [2]. Our results demonstrated that FT3 and FT4 levels were negatively correlated with the duration of HIV infection and positively correlated with the CD4 cell count. We also found higher FT3 level in patients of CDC class 1 than in patients of CDC class 3. But no relevance was found between TSH level and any of above disease parameters. In our study, CD4 cell count was lower in patients with overt hypothyroidism than in those without overt hypothyroidism, indicating that FT3 and FT4 levels were related to the progression of HIV infection to some degree. However, both univariate and multivariate logistic regression models indicated that although duration of HIV infection and CD4 cell count nadir were related to thyroid dysfunction, they were not risk factors of thyroid dysfunction. Females were at higher risk to suffer from thyroid dysfunction, which was in accordance with a recent study conducted by Sharma et al. [36]. Further studies with larger samples are needed to verify our conclusion.

Coinfection with HBV/HCV is an important factor influencing HIV progression and the effects of HAART on immune response, liver function, endocrine function, and so forth [1]. We therefore analyzed the effects of HBV/HCV coinfection on thyroid function in HIV-infected patients. We found that the levels of FT3 and FT4 were significantly increased in patients with HCV coinfection and decreased in patients with HBV coinfection. This contradicts the result reported by Beltran et al. that HCV coinfection in HIV patients is a risk factor for hypothyroidism [22]. The mechanism, via which HBV/HCV coinfection influences thyroid function, remains unclear. HBV/HCV-related autoimmune disease or side effects of interferon treatment may be potential explanations. However, only eight patients were coinfected with HCV in our study, and further studies with more HIV patients coinfected with HBV/HCV are necessary.

It is still unclear whether the cause of thyroid dysfunction in HIV patients is the HIV infection itself, its complications, therapy, or progression [2, 37, 38]. As thyroid dysfunction in HIV has been reported before the introduction of HAART, some authors have suggested that the thyroid changes are a result of opportunistic infections, regional tumors, serious systemic disease, or energy deprivation [2, 22, 39–42]. The decreased secretion of thyroid hormones may act as a form of self-protection because it decreases energy expenditure [14, 43]. However, Bongiovanni et al. concluded that therapy had an acute influence on thyroid function because patients who had recently started HAART showed a higher incidence of subclinical hypothyroidism than those who had been on HAART for at least 1 year [1, 14, 20]. Furthermore, Silva et al. suggested that immune reconstitution was more likely to protect thyroid function than impair it, and specific antiviral agents or disease activities, rather than immunity, mediated thyroid dysfunction in most AIDS patients [14].

Although the mechanism of thyroid dysfunction in HIV-infected patients is unclear, our study indicated that HIV-infected patients, particularly those on HAART, have a high incidence of thyroid dysfunction. The most common thyroid dysfunction among HIV-infected patients was hypothyroidism. Our research showed that FT3 and FT4 levels were negatively correlated with the duration of HIV infection and positively correlated with CD4 cell count in HIV-infected patients. Both these findings indicate that FT3 and FT4 levels tend to decrease with the progression of HIV infection. Therefore, physicians should pay more attention to the detection of thyroid dysfunction in HIV-infected patients and provide timely treatment. Although there were no correlations between thyroid hormone levels and duration of HAART, we demonstrated that HAART was an important factor affecting the incidence of thyroid dysfunction. Simultaneously, the influence of drugs on thyroid function should be taken into account when starting a HAART regimen or treating HBV/HCV coinfection.

Our results should be interpreted within the study's limitations: first, the sample was insufficient, especially, the number of patients with HCV coinfection. Second, drug resistance was inevitable as a result of the patients' long course of infection. The patients' medication histories were complicated as indispensable drug adjustments were performed on account of realistic clinical treatment requirements, which added to the difficulty in determining the exact influence of the drugs. Finally, this research was a retrospective cross-sectional study without follow-up to identify dynamic changes in the patients' thyroid function. A dynamic, prospective study may further clarify the relationship between HIV infection, HAART, coinfection, and thyroid function.

## Figures and Tables

**Table 1:** Demographic and clinical characteristics of 178 HIV patients.

Parameter	HAART	Naïve	Total	*P* value
Number	104 (58.4%)	74 (41.6%)	178	
Age (years)	50.18 ± 10.19	46.68 ± 11.64	48.72 ± 10.92	0.34
Sex				0.829
Males (*n*)	90 (86.5%)	63 (85.1%)	153	
Females (*n*)	14 (13.5%)	11 (14.9%)	25	
HIV transmission category				0.368
Blood	1 (1.0%)	1 (1.4%)	2	
IVDU	2 (1.9%)	1 (1.4%)	3	
MSM	69 (66.3%)	47 (63.5%)	116	
Sexual transmitted	16 (15.4%)	18 (24.3%)	34	
Unknown	16 (15.4%)	7 (9.5%)	23	
CDC stage				<0.001
A	57 (54.8%)	19 (25.7%)	76 (42.7%)	
B	37 (35.6%)	31 (41.9%)	68 (38.2%)	
C	10 (9.6%)	24 (32.4%)	34 (19.1%)	
log⁡VL	1.06 ± 1.64	4.12 ± 1.82	2.33 ± 2.29	<0.001
CD4	547 ± 305	392 ± 319	483 ± 319	0.001
CD4 nadir	371 ± 225	293 ± 220	339 ± 226	0.499
HIV infection duration (years)	14.81 ± 7.21	7.81 ± 5.26	11.90 ± 7.33	<0.001
Thyroid function				0.037
Normal	63 (60.6%)	56 (75.7%)	119 (66.9%)	
Abnormal	41 (39.4%)	18 (24.3%)	59 (33.1%)	
Subhypothyroidism	9 (8.7%)	3 (4.1%)	12 (6.6%)	
Overt hypothyroidism	28 (26.9%)	12 (16.2%)	40 (22.1%)	
Overt hyperthyroidism	4 (3.8%)	3 (4.1%)	7 (3.9%)	

HAART, HIV patients receiving highly active antiretroviral therapy (HAART); naïve: HAART naïve HIV patients; IVDU, intravenous drug user; MSM, men who have sex with men; VL: viral load.

**Table 2:** Correlation between thyroid hormone levels and HIV infection.

	*R* value	*P* value
FT3 and HIV duration	−0.221	0.004
FT4 and HIV duration	−0.213	0.006
CD4 and FT3	0.230	0.003

**Table 3:** Univariate and multivariate logistic regression models.

	Odds ratio	95% CI		*P* value
		lower	upper	
Univariate logistic regression				
Sex	2.082	0.884	4.903	0.093
HIV infection duration	1.047	1.003	1.093	0.037
CD4 nadir	0.999	0.997	1.000	0.061

Multivariate logistic regression				
Sex	2.231	0.919	5.414	0.076
HIV infection duration	1.057	1.011	1.105	0.014
CD4 nadir	0.998	0.997	1.000	0.041
